# Relationship between Rate-Limiting Process and Scaling Law in Gel Growth Induced by Liquid-Liquid Contact

**DOI:** 10.3390/gels9050359

**Published:** 2023-04-24

**Authors:** Takao Yamamoto

**Affiliations:** Division of Pure and Applied Science, Graduate School of Science and Technology, Gunma University, Kiryu 376-8515, Japan; tyam@gunma-u.ac.jp

**Keywords:** gel growth dynamics, liquid-liquid contact, rate-limiting process, free-energy-limited process, diffusion-limited process, crossover, scaling law

## Abstract

Gelation through the liquid-liquid contact between a polymer solution and a gelator solution has been attempted with various combinations of gelator and polymer solutions. In many combinations, the gel growth dynamics is expressed as $X∼\sqrt{t}$, where X is the gel thickness and t is the elapsed time, and the scaling law holds for the relationship between X and t. In the blood plasma gelation, however, the crossover of the growth behavior from X∼t in the early stage to $X∼\sqrt{t}$ in the late stage was observed. It was found that the crossover behavior is caused by a change in the rate-limiting process of growth from the free-energy-limited process to the diffusion-limited process. How, then, would the crossover phenomenon be described in terms of the scaling law? We found that the scaling law does not hold in the early stage owing to the characteristic length attributable to the free energy difference between the sol-gel phases, but it does in the late stage. We also discussed the analysis method for the crossover in terms of the scaling law.

## 1. Introduction

In the first-order phase transition, one phase becomes unstable or metastable, and a new stable phase appears owing to a temperature change [1,2,3,4]. The phase transition dynamics explain how the stable phase is created and grows. The late stage of the phase transition dynamics is visualized through the growth behavior of a small stable-phase domain in the unstable or metastable phase. The growth behavior of the stable-phase domain is described by the motion of the interface between the stable and unstable (or metastable) phases [4,5,6,7,8,9].

By adding cross-linkers to a polymer solution, a polymer network is formed, and the polymer solution is transformed into a gel [10,11,12,13,14]. Let us pay attention to physical gelation. Physical gelation is caused by the destabilization of the sol phase and the stabilization of the gel phase of the polymer solution due to the cross-linkers. If the temperature change is replaced by the addition of a cross-linker, the gelation can be viewed as a first-order phase transition. This idea leads to the expectation that the gelation process can be analyzed by focusing on the motion of the sol-gel interface. In gelation where the cross-linkers are homogeneously mixed with the polymer solution, the sol-gel interface cannot be clearly observed. Hence, it is not possible to use analysis methods that focus on the interface motion. The heterogeneous mixing of the polymer solution and the cross-linker solution through liquid-liquid contact [15] leads to gelation with a distinct sol-gel interface. Therefore, the dynamics of such gelation through the liquid-liquid contact between the cross-linker solution and the polymer solution can be analyzed by investigating the motion of the interface [16,17].

Gelation through the liquid-liquid contact between a cross-linker solution and a polymer solution has been attempted with various combinations of cross-linker and polymer solutions [15,16,17,18,19,20,21,22,23,24,25,26,27,28,29,30,31,32,33,34,35,36,37,38,39]. First, the gelation dynamics of a curdlan solution in contact with a CaCl_2_ solution [17] and then that of DNA solutions in contact with CoCl_2_ [26] and AlCl_3_ [28] solutions were investigated. The dynamics of gelation of alginate [27] and carboxymethylcellulose [29] solutions by ionic cross-linking were also observed. A distinct sol-gel interface appears in these gelations, and the motion of the interface is well described by a simple theory called the moving boundary (MB) picture [17,35], which is based on non-equilibrium thermodynamics. In the MB picture, it is assumed that the sol state becomes unstable with the influx of the cross-linker, and the polymer solution immediately gels upon the influx of the cross-linker. The MB picture shows that gelation proceeds in a diffusion-limited process; therefore, the distance between the liquid-liquid contact surface and the sol-gel interface, $X_{G}$ (gel thickness), is proportional to the square root of the elapsed time t from the start of liquid-liquid contact in the early stage. The gelation dynamics of simple systems, in which polymers are directly cross-linked by divalent metal ions, are well explained by the MB picture and expressed as $X_{G}∼\sqrt{t}$ in the early stage.

Gelators do not necessarily always directly cross-link polymer chains, such as divalent metal ions. Chitosan solution gelates upon a change in pH caused by its contact with NaOH solution. In this case, the solution with high pH is the gelator [40,41,42]. However, in the gelation of chitosan solution, not only the influx of sodium ions but also the outflow of acetic acid from the chitosan solution must be considered [40,41].

Blood coagulation is regarded as a gelation process caused by contact between blood and blood coagulation factors (initiators) [38,42,43,44,45,46,47]. The gelation of blood plasma was analyzed from the viewpoint of the gelation induced by the liquid-liquid contact. Blood gelation is a complex phenomenon involving not only diffusion but also a cascade of enzymatic reactions. The time development of the sol-gel interface in the plasma gel growth induced by the liquid-liquid contact is the result of complex processes [48]. In the gelation induced by the liquid-liquid contact, the complex processes are summed up in the dynamics of gel growth. For the plasma gelation in a rectangular cell, the linear gel growth behavior $X_{G}∼t$ in the early stage was observed. The crossover from $X_{G}∼t$ in the early stage to $X_{G}∼\sqrt{t}$ in the late stage was also observed [47].

To explain the crossover phenomenon theoretically, Dobashi and Yamamoto [38,47] introduced the Landau free energy [49] for plasma as a function of the degree of gelation. They considered that the state of plasma is changed by the inflowing gelator, and the change of the state makes the sol phase metastable and the gel phase stable. The change of the state by the inflowing gelator was called activation. They expressed the activation by the change of the functional form of the Landau free energy. The transition from the metastable sol phase to the stable gel phase in the activated plasma was described by the Ginzburg–Landau (GL) equation [50,51] based on the Landau free energy. In their proposed theory, the gelation of plasma is described as a sequential process consisting of the activation induced by the inflowing gelator and the subsequent relaxation induced by the free energy difference between the sol structure and the gel structure of the activated plasma. In the early stage of gelation, the relaxation process induced by the free energy difference is the rate-limiting process. The free-energy-limited process gives the gel growth behavior expressed as $X_{G}∼t$. In the late stage, the activation induced by the gelator diffusion is the rate-limiting process. The diffusion-limited process gives the gel growth behavior $X_{G}∼\sqrt{t}$. Hence, in their theory, the crossover behavior is due to the change in the rate-limiting process. Their theory also shows that the gel growth behavior in the early stage provides information on the thermodynamic properties of the activated plasma and that in the late stage provides that on the diffusion properties of the gelator, independently.

Their theory is a general theory, including the MB picture as its special case. This means that we can expect to find the crossover behavior in gelation processes other than plasma gelation if the experimental results of gel formation induced by the liquid-liquid contact are carefully analyzed. However, the small gel thickness in the early stage makes it difficult to accurately measure the gelation dynamics.

The scaling law was first discovered in the analysis of cardran gel growth and holds without exception in diffusion-limited liquid-liquid contact gelation [17]. The scaling law is explained as follows. Let the polymer solution be sealed in the cylindrical cell with the base radius, R, and the cell be immersed in a gelator solution. In the diffusion-limited gel growth, the time development of gel thickness can be expressed by a radius-independent function in terms of the scaled gel thickness, ${\tilde{X}}_{G}=X_{G}/R,$ and the scaled elapsed time, $\tilde{t}=t/R^{2}$. The scaling law holds since there is no length scale characterizing the system other than the radius, R. In the free-energy-limited growth, the scaling by the radius does not hold since there is a characteristic length scale due to the free energy. In the gel growth dynamics, where the crossover occurs, the scaling law does not hold in the early stage, but it does in the late stage. In the present article, from the viewpoint of scaling, we analyze the crossover phenomenon of rate-limiting processes of the liquid-liquid contact induced gelation.

## 2. Theoretical Model

**Figure 1:**
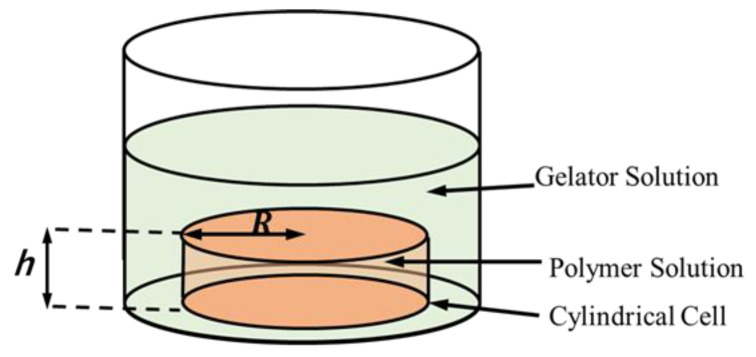
Cylindrical cell containing polymer solution immersed in gelator solution. The polymer solution is encapsulated in a cylindrical cell with a base radius, R, and height, h, by sealing its side with a dialysis membrane.

To analyze the scaling in the gelation, let us consider the polymer solution sealed in the cylindrical cell with the base radius, R, and height, h, as shown in Figure 1. The side of the cylindrical cell is made of a dialysis membrane. The cell is immersed in a gelator solution. Gelators can flow into the polymer solution in the cell through the membrane from the side of the cell. In the present article, we focus on the relationship between gelation dynamics and the characteristic length, i.e., the radius, R.

**Figure 2:**
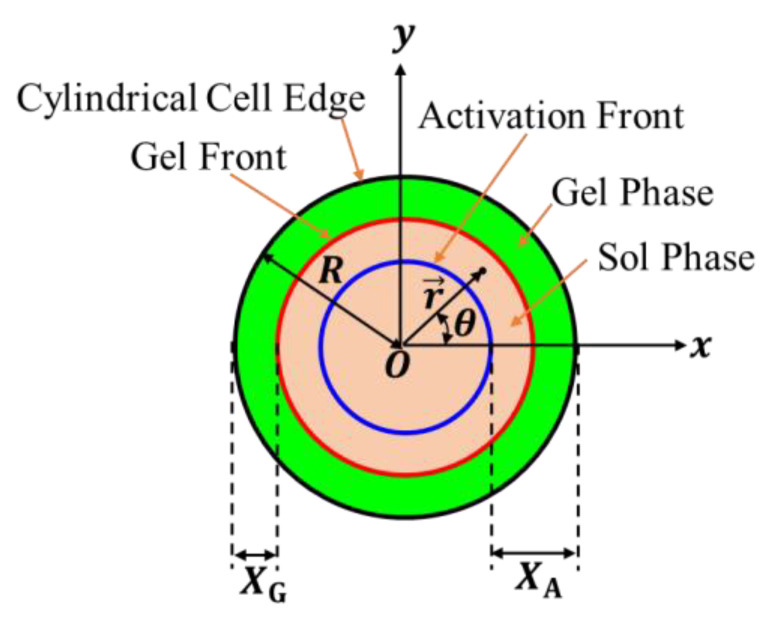
Coordinate system and variables used to describe the gelation dynamics. The x–y plane is located on a basal plane of the cylindrical cell so that its origin coincides with the center of the basal plane. The distances between the edge of the cylindrical cell and the activation and gel fronts are respectively denoted by $X_{A}$ and $X_{G}$.

We choose the x–y plane so that a basal plane is located on it, and the center of the basal plane coincides with the origin (see Figure 2). The unit vectors along the x- and y-directions are respectively denoted by ${\vec{e}}_{x}$ and ${\vec{e}}_{y}$. For convenience, we choose the polar coordinate $\left(r,\theta \right)$, where θ is the angle between the x-axis and the position vector $\vec{r}=x{\vec{e}}_{x}+y{\vec{e}}_{y}$, and r is the distance from the origin ($r=\left|\vec{r}\right|$). The unit vector along the radial direction is given by ${\vec{e}}_{r}=\cos\theta {\vec{e}}_{x}+\sin\theta {\vec{e}}_{y}$.

The polymer solution in the cylindrical cell is gelled by the inflowing gelator from the gelator solution. Let us assume that the gelation consists of the following two processes occurring in sequence [38]. From now on, let this idea be called “the sequential picture”.

Process I: The gelators bind to the gelation points of polymer chains in the polymer solution and “activate” the gelation points of polymer chains.

Process II: The polymer chains with activated gelation points bind together to form a gel.

For Process I, we make the following two assumptions regarding the flow of the gelator and the activation of the polymer solution by the gelator [17].

Assumption I: The gelators flowing into the nonactivated polymer solution instantly activate the polymer chains at the inflowing point, and all of the inflowing gelators are consumed to activate the polymer solutions.

Assumption II: The activated polymer solution does not capture the inflowing gelators.

Assumption II indicates that Assumption I also requires that no nonactivated polymer chains exist in the polymer solution activated by the inflowing gelators. Assumption I ensures that the boundary between the nonactivated polymer solution and the activated polymer solution is macroscopically distinct and that the activation process of the polymer solution can be visualized by tracking the motion of the boundary. Let us call the boundary the activation front. From the symmetry of the system, it can be observed that the activation front forms a circular pattern whose center is the origin of the x–y plane. The distance of the activation front from the center at the immersion time t is denoted by $r_{A}\left(t\right)$; the polymer solution in the outer region $r_{A}\leq r\leq R$ is activated, and that in the inner region $r<r_{A}$ is not activated. The distance $X_{A}$ between the activation front and the edge of the cylindrical cell is given by

(1) $$X_{A}\left(t\right)=R-r_{A}\left(t\right).$$

The growth of the activated region is expressed as the time development of the activation front, $X_{A}$.

The gelation dynamics induced by Process II are expressed as a relaxation behavior from high- to low-free energy states. To describe the thermodynamic state of the polymer solution in the cylindrical cell, the order parameter for the degree of gelation ϕ is introduced such that the polymer solution is a gel for ϕ>0 and a sol for ϕ=0. We assume that the free energy per unit volume of the polymer solution at a homogeneous state at ϕ is given by

(2) $$f\left(\phi \right)=g\phi^{2}{\left(1-\phi \right)}^{2}+a\phi^{2},$$

where g is a positive constant and a depends on whether the polymer solution is in the activated state or not. The parameter a takes either a large value of $a_{0}$ or a small value of $a_{m}$ ($a_{0}>a_{m}$);

(3) $$a=\left\{\begin{array}{ccc} a_{0} & & \mathrm{when}\;\mathrm{the}\;\mathrm{polymer}\;\mathrm{solution}\;\mathrm{is}\;\mathrm{in}\;\mathrm{the}\;\mathrm{nonactivated}\;\mathrm{state}.\\ a_{m} & & \mathrm{when}\;\mathrm{the}\;\mathrm{polymer}\;\mathrm{solution}\;\mathrm{is}\;\mathrm{in}\;\mathrm{the}\;\mathrm{activated}\;\mathrm{state}. \end{array}\right..$$

**Figure 3:**
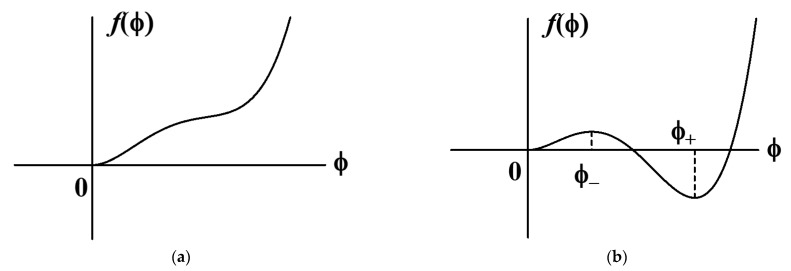
Local free energy of polymer solution for $a=a_{0}$ (**a**) and $a=a_{m}$ (**b**).

Let the local free energy function $f\left(\phi \right)$ have only the minimum value at ϕ=0 in the nonactivated state and have two minima at ϕ=0 and $\phi =\phi_{+}$ and one maximum at $\phi =\phi_{-}$ in the activated state, where $0<\phi_{-}<\phi_{+}$ (see Figure 3). Therefore, $a_{0}$ should be larger than g/8. In the activated state, the local free energy function $f\left(\phi \right)$ is required to have two minima at ϕ=0 and $\phi =\phi_{+}$ and one maximum at $\phi =\phi_{-}$, where $0<\phi_{-}<\phi_{+}$, as shown in Figure 3b. From this requirement, $a_{m}$ should satisfy the following inequality:

(4) $$\frac{g}{8}>a_{m}>-g.$$

When the condition (4) is satisfied, the following expressions are obtained:

(5) $$\frac{\partial f}{\partial \phi }=4g\phi \left(\phi -\phi_{-}\right)\left(\phi -\phi_{+}\right),$$

where

(6) $$\phi_{\pm }=\frac{3\pm \sqrt{1-\frac{8a_{m}}{g}}}{4},$$

(7) $$f\left(0\right)=0,$$

and

(8) $$f\left(\phi_{+}\right)=\frac{1}{3}g\phi_{+}^{3}\left(2\phi_{-}-\phi_{+}\right)=\frac{1}{4}g\phi_{+}^{3}\left(1-\sqrt{1-\frac{8a_{m}}{g}}\right).$$

For the one-dimensional system discussed previously [38], Process II is described by the change in ϕ from 0 to $\phi_{+}$ caused by only the free energy difference $f\left(0\right)-f\left(\phi_{+}\right)$. Therefore, gelation does not proceed when $f\left(0\right)-f\left(\phi_{+}\right)\leq 0$. For the two-dimensional cylindrical system discussed in the present article, the interface free energy between the sol and gel layers, as well as the free energy difference, also drives gelation. Therefore, even if $f\left(0\right)-f\left(\phi_{+}\right)\leq 0$, gelation proceeds. The effect of interface free energy on gelation is a characteristic of two- and three-dimensional gelation processes induced by the liquid-liquid contact process.

In the region $r<r_{A}$, the polymer solution is in a nonactivated state. Therefore $a=a\left(r\right)=a_{0}$. On the other hand, in the region $r\geq r_{A}$, $a\left(r\right)=a_{m}$. These considerations mean that the function form of the polymer solution free energy $f\left(\phi \right)$ depends on the position, $f\left(\phi \right)=f\left(\phi ,a\left(r\right)\right)$, and the degree of gelation ϕ must be considered as a function of the position $\vec{r}$. Therefore, as the total free energy of the polymer solution per unit height, we introduce the following functional:

(9) $$F=\overset{}{\underset{\left|\vec{r}\right|\leq R}{\iint }}\left[\frac{1}{2}\kappa {\left(\nabla \phi \right)}^{2}+f\left(\phi ,a\left(r\right)\right)\right]d^{2}\vec{r},$$

where $\nabla =\frac{\partial }{\partial x}{\vec{e}}_{x}+\frac{\partial }{\partial y}\;{\vec{e}}_{y}$ and κ is a small positive number.

The gelation process given by Process II is regarded as the equilibration of the degree of gelation from the sol state ϕ=0 to the gel state $\phi =\phi_{+}$. The GL equation well describes such equilibration processes [49,50,51]. Therefore, we adopt the following GL equation to describe the dynamics of the equilibration process:

(10) $$\frac{\partial \phi }{\partial t}=-\Gamma \frac{\delta F}{\delta \phi },$$

where Γ is a positive constant called the kinetic coefficient.

For the symmetry of the system, the solution of Equation (10) is a function of the distance from the origin, r, and the immersion time t; $\phi =\phi \left(r,t\right)$. As in the one-dimensional system, a kink-type solution expressing a stationary gel growth is expected:

(11) $$\phi \left(r,t\right)=\left\{\begin{array}{cc} 0 & r<r_{G}\left(t\right).\\ \phi_{+} & r_{G}\left(t\right)\leq r\leq R. \end{array}\right.$$

Therefore, the sol-gel boundary is given by $r=r_{G}\left(t\right)$. Then, the gel thickness, $X_{G}\left(t\right),$ expressing the gel growth behavior is given by

(12) $$X_{G}\left(t\right)=R-r_{G}\left(t\right).$$

## 3. Derivation of Gelation Dynamics

### 3.1. Motion of Activation Front

The dynamics of the activation front, according to Assumptions I and II, can be derived similarly to the derivation of the diffusion-limited gel growth on the basis of the MB picture [17]. Owing to the cylindrical symmetry, the inflow gelator flux, $\vec{j}\left(r\right),$ is along the radial direction and only depends on the distance r from the origin. The inflow gelator flux in the activated region $R\geq r\geq R-X_{A}$ is given by

(13) $$\vec{j}\left(r\right)=-j\left(r\right){\;\vec{e}}_{r},$$

where $j\left(r\right)$ is the flux density and ${\;\vec{e}}_{r}$ is the unit vector along the radial direction. On the basis of Assumption I, we obtain the relationship between the thickness $dX_{A}$ of the newly activated polymer region and the time interval dt as

(14) $$2\pi \left(R-X_{A}\right)j\left(R-X_{A}\right)dt=2\pi \left(R-X_{A}\right)\rho_{A}dX_{A},$$

where $\rho_{A}$ denotes the number of gelator molecules activating a unit volume of the polymer solution. Therefore, we obtain the differential equation satisfied by $X_{A}$ as

(15) $$\frac{dX_{A}}{dt}=\frac{1}{\rho_{A}}j\left(R-X_{A}\right).$$

By denoting the gelator concentration in the activated region by $\rho \left(r,t\right)$ from Assumption II, we obtain the equation of continuity.

(16) $$\frac{\partial \rho }{\partial t}+\nabla \cdot \vec{j}=0$$

Let the stationary flow of the gelator be assumed as in the original MB picture [17]. Then, we obtain $\frac{\partial \rho }{\partial t}=0$ and

(17) $$\nabla \cdot \vec{j}=0.$$

Using the cylindrical symmetry for $\vec{j}\left(r\right)$ shown in Equation (13) and the polar coordinate expression, we rewrite Equation (17) as

(18) $$-\frac{1}{r}\frac{\partial }{\partial r}\left(rj\right)=0.$$

The flux density, $j\left(r\right),$ obtained as the solution of the above equation is given by

(19) $$j\left(r\right)=\frac{C}{r},$$

where C is an integral constant determined by the boundary conditions.

To obtain the integral constant, let the flux density be related to the gelator concentration. For simplicity, we assume that the gelator concentration is low. Then, in terms of the diffusion coefficient, D, of the gelator in the activated polymer solution, the flux is expressed as

(20) $$\vec{j}=-D\nabla \rho =-D\frac{\partial \rho }{\partial r}\;{\vec{e}}_{r}.$$

By comparing Equation (20) with Equation (13), we have $j=D\frac{\partial \rho }{\partial r}$ and the rewritten form of Equation (19) as

(21) $$D\frac{\partial \rho }{\partial r}=\frac{C}{r}.$$

By integrating both sides of Equation (21) from $r=R-X_{A}$ to r=R, we obtain

(22) $$D\left[\rho \left(R\right)-\rho \left(R-X_{A}\right)\right]=C\ln\frac{R}{R-X_{A}}.$$

The polymer solution in the cylindrical cell is in contact with the gelator solution with the gelator concentration, $\rho_{s,}$ at the dialysis membrane, r=R. Then, $\rho \left(R\right)=\rho_{s}$. Assumption I shows that the gelators are absent in the nonactivated polymer solution. Then, $\rho \left(R-X_{A}\right)=0$. From these boundary conditions and Equation (22), the integral constant is given by

(23) $$C=\frac{D\rho_{s}}{\ln\frac{R}{R-X_{A}}}.$$

Hence, the time development equation of the activation front $X_{A}$ is obtained as

(24) $$\frac{dX_{A}}{dt}=K\frac{1}{\left(R-X_{A}\right)\ln\frac{R}{R-X_{A}}},$$

with

(25) $$K=D\frac{\rho_{s}}{\rho_{A}}.$$

By introducing the scaled time $\tilde{t}$ and the scaled thickness of the activated region ${\tilde{X}}_{A}$ as [17]

(26) $$\left.\begin{array}{c} \tilde{t}=\frac{t}{R^{2}}\\ {\tilde{X}}_{A}=\frac{X_{A}}{R} \end{array}\right\},$$

we obtain the following “universal” expression independent of the radius R of the cylindrical cell:

(27) $$\frac{d{\tilde{X}}_{\;A}}{d\tilde{t}}=K\frac{1}{\left(1-{\tilde{X}}_{A}\right)\ln\frac{1}{1-{\tilde{X}}_{A}}}.$$

The solution to the above equation is given by [17]

(28) $${\tilde{Y}}_{A}\left({\tilde{X}}_{A}\right)=K\tilde{t},$$

where

(29) $${\tilde{Y}}_{A}\left({\tilde{X}}_{A}\right)\equiv \frac{1}{2}{\left(1-{\tilde{X}}_{A}\right)}^{2}\ln\left(1-{\tilde{X}}_{A}\right)-\frac{1}{4}{\tilde{X}}_{A}^{2}+\frac{1}{2}{\tilde{X}}_{A}$$

is a universal function irrespective of the details of the system. Only the parameter K indicates the individuality of the activation dynamics.

### 3.2. Motion of Gel Front

The nonactivated polymer solution remains in the sol phase, whereas the activated polymer solution is gelled according to the GL equation given by Equation (10). Using expression (9), we can write the GL equation as

(30) $$-\tau \frac{\partial \phi }{\partial t}=-\kappa \nabla^{2}\phi +4g\phi \left(\phi -\phi_{-}\right)\left(\phi -\phi_{+}\right),$$

where

(31) $$\tau =\frac{1}{\Gamma }.$$

For the cylindrical symmetry, we can write $\nabla^{2}$ as

(32) $$\nabla^{2}=\frac{\partial^{2}}{\partial r^{2}}+\frac{1}{r}\frac{\partial }{\partial r},$$

and rewrite Equation (30) as

(33) $$-\tau \frac{\partial \phi }{\partial t}=-\kappa \left(\frac{\partial^{2}\phi }{\partial r^{2}}+\frac{1}{r}\frac{\partial \phi }{\partial r}\right)+4g\phi \left(\phi -\phi_{-}\right)\left(\phi -\phi_{+}\right).$$

By rewriting Equation (33) in terms of the distance from the edge of the cylindrical cell w=R−r, we obtain

(34) $$-\tau \frac{\partial \phi }{\partial t}=-\kappa \left(\frac{\partial^{2}\phi }{\partial w^{2}}-\frac{1}{R-w}\frac{\partial \phi }{\partial w}\right)+4g\phi \left(\phi -\phi_{-}\right)\left(\phi -\phi_{+}\right).$$

Note that the above equation is valid in the region $0\leq w\leq X_{A}$. The term $\kappa \frac{1}{R-w}\frac{\partial \phi }{\partial w}$ on the right-hand side shows the interface effect on gelation and is absent in the GL equation for a one-dimensional system.

Suppose that as soon as the cell is immersed in the gelator solution, gel nuclei are generated inside the dialysis membrane, sealing the side of the cylindrical cell, and a thin gel film whose thickness is negligible macroscopically forms. Hence, as the initial condition of the gelation dynamics, we assume that the polymer solution very near the dialysis membrane is in the gel state and that inside, it is in the sol state.

According to Chan [6] and Allen and Cahn [7], we can obtain the stationary solution of Equation (34) as

(35) $$\phi \left(w,t\right)=\psi \left(w-X_{G}\left(t\right)\right),$$

where

(36) $$\psi \left(w\right)=\frac{\phi_{+}}{1+e^{w/\lambda }}$$

with

(37) $$\lambda =\frac{1}{\phi_{+}}\sqrt{\frac{\kappa }{2g}}.$$

The function $X_{G}\left(t\right)$ is given as the solution to the equation

(38) $$\frac{dX_{G}}{dt}=\frac{V_{0}}{R-X_{G}}+\frac{V_{0}}{\xi },$$

where

(39) $$\xi =\frac{\phi_{+}}{\phi_{+}-2\phi_{-}}\lambda =\frac{4}{3}\frac{\phi_{+}}{\sqrt{1-\frac{8a_{m}}{g}}-1}\lambda =\frac{\phi_{+}^{4}}{3}\frac{g}{f\left(0\right)-f\left(\phi_{+}\right)}\lambda$$

and

(40) $$V_{0}=\frac{\kappa }{\tau }=\frac{2g\phi_{+}^{2}\lambda^{2}}{\tau }.\;$$

The solution (36) is a kink-type function connecting the gel state $\phi \left(w,t\right)=\phi_{+}$ and the sol state $\phi \left(w,t\right)=0$, and the length λ in the solution is the thickness of the boundary between the sol and gel states. For the boundary to be clearly visible, the boundary should be macroscopically very narrow; then, R≫λ. The length λ is regarded as the smallest unit of length for the macroscopic view. In the macroscopic view in which the length scale is much larger than λ, the state of the polymer solution changes markedly at $w=X_{G}$ and the gel front position is given by $w=X_{G}$. The initial condition for the gelation dynamics can be rewritten as the initial condition $X_{G}\left(0\right)=0$ for the dynamics of the gel front $X_{G}\left(t\right)$ given by Equation (38).

From Equation (38), it is found that for the gel front to move forward, the following inequality should be satisfied:

(41) $$\frac{1}{R}+\frac{1}{\xi }>0.$$

This inequality and the expression (39) show that the condition that the free energy is minimum in the gel state $\phi =\phi_{+}$, $f\left(0\right)>f\left(\phi_{+}\right)$, is not necessarily required for gelation. For a one-dimensional system, however, the condition $f\left(0\right)>f\left(\phi_{+}\right)$ is required for the gel to grow [38]. The difference lies in the dependence of the interface free energy on the gel thickness; in a one-dimensional system, the interface free energy is independent of the gel thickness, but it is dependent on the cylindrical system. To confirm this dependence, let us evaluate the interface free energy part in the total free energy F. The interface free energy is evaluated as

(42) $$F_{I}=\overset{}{\underset{\left|\vec{r}\right|\leq R}{\iint }}\left[\frac{1}{2}\kappa {\left(\nabla \psi \left(r-\left(R-X_{G}\right)\right)\right)}^{2}\right]d^{2}\vec{r}\;\;\;\;\;\;=\int_{0}^{R}\frac{g\phi_{+}^{4}}{{\left(e^{-\frac{1}{2\lambda }\left(r-\left(R-X_{G}\right)\right)}+e^{\frac{1}{2\lambda }\left(r-\left(R-X_{G}\right)\right)}\right)}^{4}}2\pi rdr\;\;\;\;≃\int_{R-X_{G}-2\lambda }^{R-X_{G}+2\lambda }\frac{g\phi_{+}^{4}}{16}2\pi rdr=\frac{\pi }{2}g\lambda \phi_{+}^{4}\left(R-X_{G}\right)$$

The above evaluation shows that $F_{I}$ is a decrease function of $X_{G}$. The interface free energy, $F_{I,}$ is an increase function of the area of the interface since the area per unit height of the interface is given by $2\pi \left(R-X_{G}\right)$. The gel front moves forward to decrease the interface free energy when the decrease in interface free energy induced by the gel growth outweighs the local free energy loss. Since the area of the interface is independent of the position of the gel front in a one-dimensional system, i.e., the interface free energy is independent of the position of the gel front, the condition $f\left(0\right)>f\left(\phi_{+}\right)$ for the free energy difference between the sol and gel phases is required for the gel to grow. The first term $V_{0}/\left(R-X_{G}\right)$ and the second term $V_{0}/\xi$ on the right-hand side of Equation (38) are respectively the driving forces for gelation due to the interface free energy and the free energy difference.

As in the case of the activation front motion, we introduce the scaled variables as

(43) $$\left.\begin{array}{c} \tilde{t}=\frac{t}{R^{2}}\\ {\tilde{X}}_{G}=\frac{X_{G}}{R} \end{array}\right\},$$

and we rewrite Equation (38) in terms of the scaled variables as

(44) $$\frac{d{\tilde{X}}_{G}}{d\tilde{t}}=V_{0}\frac{1}{1-{\tilde{X}}_{G}}+V_{0}\frac{R}{\xi }.$$

When ξ is finite, unlike in the motion of the activation front, the time development equation for the gel front given by the above equation is not invariant to the scale transformation Equation (43) owing to the presence of the radius-dependent term R/ξ. However, if ξ=∞, i.e., $f\left(0\right)=f\left(\phi_{+}\right)$, then the radius-dependent term is absent, and the time development equation is invariant to the scale transformation.

When ξ is finite, the solution of the above equation for the initial condition ${\tilde{X}}_{G}\left(0\right)=0$ is given by

(45) $$Q\left({\tilde{X}}_{G}\right)=V_{0}\tilde{t},$$

where

(46) $$Q\left({\tilde{X}}_{G}\right)=\frac{\xi }{R}{\tilde{X}}_{G}+{\left(\frac{\xi }{R}\right)}^{2}\ln\left[1-\frac{1}{1+\xi /R}{\tilde{X}}_{G}\right].$$

Let us consider the case ξ=∞, that is, consider the case $f\left(0\right)=f\left(\phi_{+}\right)$. The time development Equation (44) for ${\tilde{X}}_{G}$ is expressed as

(47) $$\frac{d{\tilde{X}}_{G}}{d\tilde{t}}=V_{0}\frac{1}{1-{\tilde{X}}_{G}}.$$

The above equation is invariant to the scale transformation Equation (43). Then, the scale transformation invariant solution is obtained as

(48) $$Q_{0}\left({\tilde{X}}_{G}\right)=V_{0}\tilde{t},$$

with

(49) $$Q_{0}\left({\tilde{X}}_{G}\right)={\tilde{X}}_{G}-\frac{1}{2}{\tilde{X}}_{G}^{2}.$$

Note that

(50) $$\underset{\frac{\xi }{R}\to \infty }{\lim}Q\left({\tilde{X}}_{G}\right)=Q_{0}\left({\tilde{X}}_{G}\right).$$

## 4. Discussion: Crossover and Scaling

The time development of the scaled gel front, ${\tilde{X}}_{G,}$ is expressed as Equation (45) when the following inequality is satisfied:

(51) $$0\leq {\tilde{X}}_{G}\leq {\tilde{X}}_{A},$$

where the time development of the scaled activation front, ${\tilde{X}}_{A,}$ is given by Equation (26). In the early stage $\tilde{t}≃0$, Equations (28) and (45), respectively, give the initial behaviors for ${\tilde{X}}_{A}$ and ${\tilde{X}}_{G}$ as ${\tilde{X}}_{A}≃\sqrt{2K\tilde{t}}$ and ${\tilde{X}}_{G}≃V_{0}\left(1+R/\xi \right)\tilde{t}$. Therefore, the inequality (51) is satisfied since $\frac{\tilde{t}}{\sqrt{\tilde{t}}}≃0$ in the early stage. Since the scaled velocity of the scaled gel front, $d{\tilde{X}}_{G}/d\tilde{t,}$ exceeds that of the scaled activation front, $d{\tilde{X}}_{A}/d\tilde{t,}$ as time elapses, the gel front could catch up with the activation front [47]. The gel front must move with the activation front after the gel front catches up with the activation front. Hence, the gel front motion changes at which the gel front catches up with the activation front from the free-energy-limited motion derived from the GL Equation (10) to the diffusion-limited motion dominated by gelator diffusion; the crossover behavior of the gel front motion appears [47].

Let us discuss the crossover behavior in the case of $f\left(0\right)-f\left(\phi_{+}\right)>0$. The crossover behavior appears when the two curves $\tilde{t}={\tilde{Y}}_{A}\left(\tilde{X}\right)/K$ and $\tilde{t}=Q\left(\tilde{X}\right)/V_{0}$ on the $\left(\tilde{X},\tilde{t}\right)$ plane cross in the region $0<\tilde{X}<1$. Since $\frac{{\tilde{Y}}_{A}\left(\tilde{X}\right)}{K}≃\frac{{\tilde{X}}^{2}}{2K}$ and $\frac{Q\left(\tilde{X}\right)}{V_{0}}≃V_{0}^{-1}\frac{\xi }{R+\xi }\tilde{X}$ for small $\tilde{X}$, we have the inequality $\frac{Q\left(\tilde{X}\right)}{V_{0}}>\frac{{\tilde{Y}}_{A}\left(\tilde{X}\right)}{K}$ near $\tilde{X}=0$. Therefore, the two curves cross if $\frac{Q\left(\tilde{X}\right)}{V_{0}}<\frac{{\tilde{Y}}_{A}\left(\tilde{X}\right)}{K}$ near $\tilde{X}=1$. Hence, the condition under which the crossover occurs is $\frac{Q\left(1\right)}{V_{0}}<\frac{{\tilde{Y}}_{A}\left(1\right)}{K},$ and the condition is written as

(52) $$K<K_{FL},\;$$

with

(53) $$K_{FL}=\frac{{\tilde{Y}}_{A}\left(1\right)V_{0}}{Q\left(1\right)}=\frac{1}{4}V_{0}\frac{R}{\xi }\;\frac{1}{1-\frac{\xi }{R}\ln\left(1+\frac{R}{\xi }\right)}.\;$$

The “crossover time,” $t_{c,}$ at which the gel front motion changes are obtained from the following simultaneous equations with respect to the scaled crossover time ${\tilde{t}}_{c}=t_{c}/R^{2}$ and the scaled gel thickness, ${\tilde{X}}_{c,}$ at the crossover time:

(54) $$\frac{Q\left({\tilde{X}}_{c}\right)}{V_{0}}=\frac{{\tilde{Y}}_{A}\left({\tilde{X}}_{c}\right)}{K}={\tilde{t}}_{c}.\;$$

The motion change is expressed by the following change of the function form expressing the motion:

(55) $$\left\{\begin{array}{cc} Q\left({\tilde{X}}_{G}\right)=V_{0}\tilde{t}, & \tilde{t}\leq {\tilde{t}}_{c}\\ {\tilde{Y}}_{A}\left({\tilde{X}}_{G}\right)=K\tilde{t}, & \tilde{t}>{\tilde{t}}_{c} \end{array}\right..$$

In the early part of gelation, $0\leq \tilde{t}\leq {\tilde{t}}_{c}$, the gel grows in the free-energy-limited process, and the growth behavior is expressed by the function Q. In the latter part of gelation ${\tilde{t}}_{c}<\tilde{t}$, the gel grows in the diffusion-limited process, and the growth behavior is expressed by the function ${\tilde{Y}}_{A}$.

**Figure 4:**
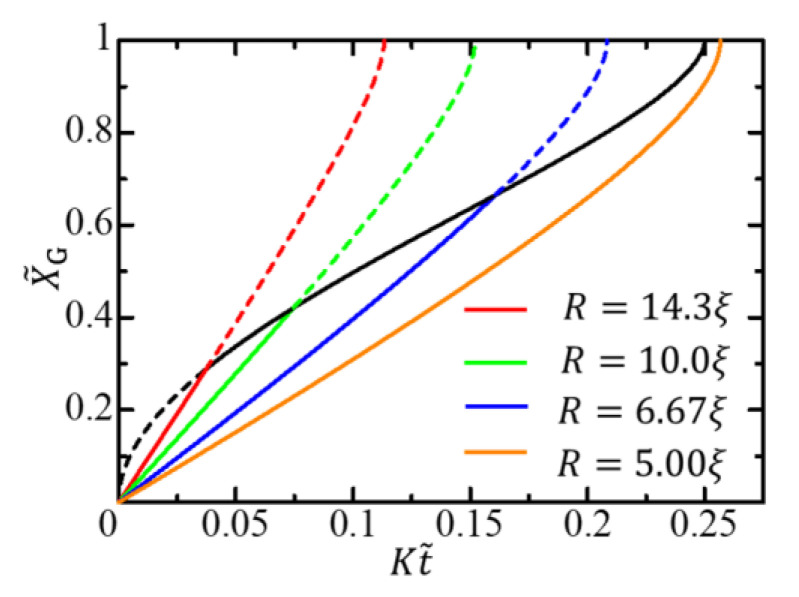
Gel growth behavior expressed by ${\tilde{X}}_{G}$–$\tilde{t}$ curves for different radii. The red, green, blue, and orange solid curves, respectively, show the growth behaviors for R=14.3ξ, R=10.0ξ, R=6.67ξ, and R=5.00ξ in the free-energy-limited time region, where $V_{0}=0.500K$. The black solid curve shows the growth behavior in the diffusion-limited time region.

The function ${\tilde{Y}}_{A}$ is independent of the radius R. Therefore, in terms of the scaled variables given by Equation (43), the gel growth curve is independent of the radius in the diffusion-limited growth time region. In contrast, in the free-energy-limited growth time region, the curve depends on the radius since the function Q depends on the radius. The ${\tilde{X}}_{G}$–$\tilde{t}$ curves for different radii are initially different curves depending on the radius but converge to a single curve in the late stage, as shown in Figure 4. The change from the radius-dependent gel-growth curve to the radius-independent gel-growth curve characterizes the crossover behavior from the free-energy-limited growth to the diffusion-limited growth and facilitates the experimental observation of the crossover behavior.

When $f\left(0\right)-f\left(\phi_{+}\right)>0$, the quantity ξ is regarded as a characteristic length attributable to the free energy difference between the sol and gel phases. In the free-energy-limited growth, there are two characteristic lengths ξ and R. Therefore, the growth behavior cannot be scaled by the radius R. In the diffusion-limited growth, however, the radius R is the only characteristic length scale. Hence, the gel growth behavior scaled by the radius R is described by the radius-independent function ${\tilde{Y}}_{A}$.

When the free-energy-limited growth is slow, the diffusion-limited growth process does not appear. In this case, the activation front reaches the center of the cylindrical cell before the gel front catches up with the activated front, and the gel growth proceeds only through the free-energy-limited process. The condition under which the diffusion-limited growth does not appear is $K\geq K_{FL}$. The condition is satisfied not only when the free-energy-limited growth is slow but also when R is small. Therefore, for cells with small radii, the entire gel growth process is free energy-limited (the orange solid curve in Figure 4).

The properties of the activated polymer solution can be investigated in terms of the radius dependence of the gel growth rate. Equation (38) shows that in the early stage, the rate of increase in gel thickness is independent of elapsed time and is a function of the radius R as follows.

(56) $$\frac{dX_{G}}{dt}=V_{0}\left(\frac{1}{R}+\frac{1}{\xi }\right)\;$$

By measuring the rate of increase in gel thickness in cells with different radii, we obtain the two parameters, $V_{0}$ and ξ, characterizing the activated polymer solution. Hence, by the scaling analysis, all the parameters K, $V_{0}$ and ξ that determine the progress of gelation are obtained. This means that the gelation progression can be controlled.

Next, the case of $f\left(0\right)-f\left(\phi_{+}\right)=0$ is discussed. In this case, gelation does not proceed spontaneously, even if the polymer solution is activated by the influx of the gelator. The gel film of macroscopically negligible thickness on the dialysis membrane, which is necessary for the initial condition of the gelation dynamics, does not form spontaneously. The gel film must be formed on the dialysis membrane in advance.

In this case, the characteristic length attributable to the free energy difference disappears, and the cell radius R is the only characteristic length in the free-energy-limited growth. The free-energy-limited growth behavior is expressed by $Q_{0}$. The function $Q_{0}$ has no parameters characterizing the system at all, not just the radius R. The coefficient $V_{0}$ is the only parameter characterizing the free-energy-limited growth. The crossover condition is independent of the radius R and is expressed as

(57) $$K<\frac{1}{2}V_{0}\;$$

**Figure 5:**
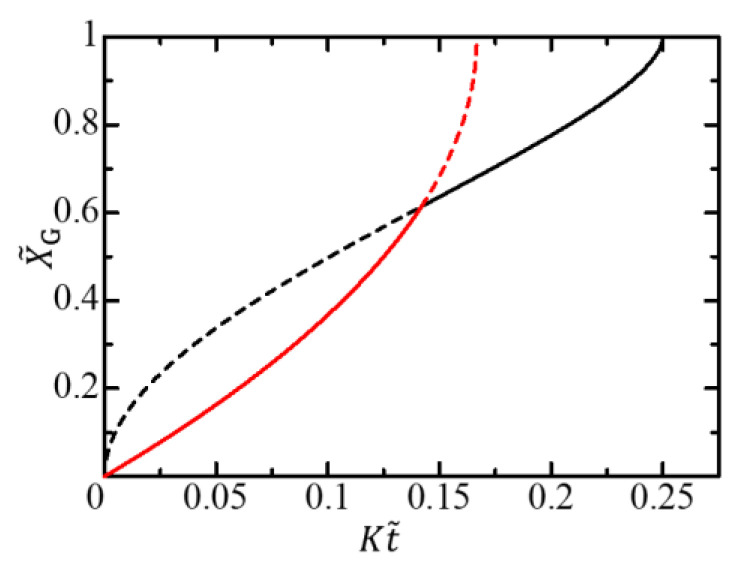
Gel growth behavior expressed by the ${\tilde{X}}_{G}$–$\tilde{t}$ curve for a large ξ limit. The red solid curve shows the growth behavior in the free-energy-limited time region, where $V_{0}=3.00K$. The black solid curve shows the growth behavior in the diffusion-limited time region. The curves are independent of the radius R.

The scaled crossover time ${\tilde{t}}_{c}$ and the scaled crossover thickness ${\tilde{X}}_{c}$ are also independent of the radius R. An example of an ${\tilde{X}}_{G}$–$\tilde{t}$ curve is shown in Figure 5. The gel growth curve is invariant to the scale transformation Equation (43). Therefore, we cannot find any crossover from the R-dependence of the gel growth curve.

Even if $f\left(0\right)-f\left(\phi_{+}\right)$ is not exactly zero but is a sufficiently small positive value, i.e., when R≪ξ, the function Q can be regarded as the function $Q_{0}$. Hence, when ξ is sufficiently large if the crossover appears, the gel growth behavior is practically scale-transformation-invariant. It would take time for a thin gel film necessary for the initial condition of the gel dynamics to form on the dialysis membrane. Hence, a lag time would be observed before gel growth begins.

When $f\left(0\right)-f\left(\phi_{+}\right)<0$, ξ is negative. For a negative ξ, the time development equation in the early stage Equation (56) still holds. However, for the equation to be meaningful as the equation for gel growth, the radius R should be smaller than $\left|\xi \right|$, and a gel layer must be formed previously on the dialysis membrane as the initial condition. In this case, the gel phase is metastable, not stable. Gelation is driven by interface free energy, and the free energy difference between the sol and gel phases rather inhibits gel growth. The crossover condition is given by Equation (52) with Equation (53) for a negative  ξ.

Finally, let us consider the case where the entire gel growth process is diffusion-limited from the viewpoint of the sequential picture. The first idea is that the observation of the free-energy-limited growth in the early stage is missed because it appears only for a very short time. In fact, it is difficult to accurately measure the gel thickness in the early stages of gelation. It would be difficult to establish that the gel growth is free-energy-limited on the basis of only the data measured during a short period of time in the early stages of gelation. However, the scaling-based analysis proposed in the present article may enable the finding of the short-time free-energy-limited gel growth process. Even in the case of cross-linking by multivalent metal ions, the crossover phenomenon may be observed.

One of the other possible scenarios is when there is no maximum in the free energy of the activated polymer solution and the sol state is unstable, and the gel state is the only stable state. In this case, the equilibration process expressing Process II is given by

(58) $$\frac{\partial \phi \left(r,t\right)}{\partial t}=-\frac{1}{\tau^{\prime }}\left(\phi \left(r,t\right)-\phi_{eq}\left(r,t\right)\right)\;$$

with the initial condition $\phi \left(r,0\right)=0$. In the above, $\tau^{\prime }$ is a positive time constant and

(59) $$\phi_{\mathrm{eq}}\left(r,t\right)=\left\{\begin{array}{cc} 0 & r<r_{A}\left(t\right)\\ \phi_{+} & r_{A}\left(t\right)\leq r\leq R \end{array}\right..\;$$

From Equation (58), the gel front is obtained as

(60) $$X_{G}\left(t\right)=\left\{\begin{array}{cc} 0 & t<\tau^{\prime }\\ X_{A}\left(t-\tau^{\prime }\right) & \tau^{\prime }\leq t \end{array}\right..\;$$

Since the motion of the gel front follows that of the activation front except for a delay of only a short relaxation time $\tau^{\prime }$, the gel proceeds in the diffusion-limited process; in this scenario, the crossover phenomenon does not appear.
